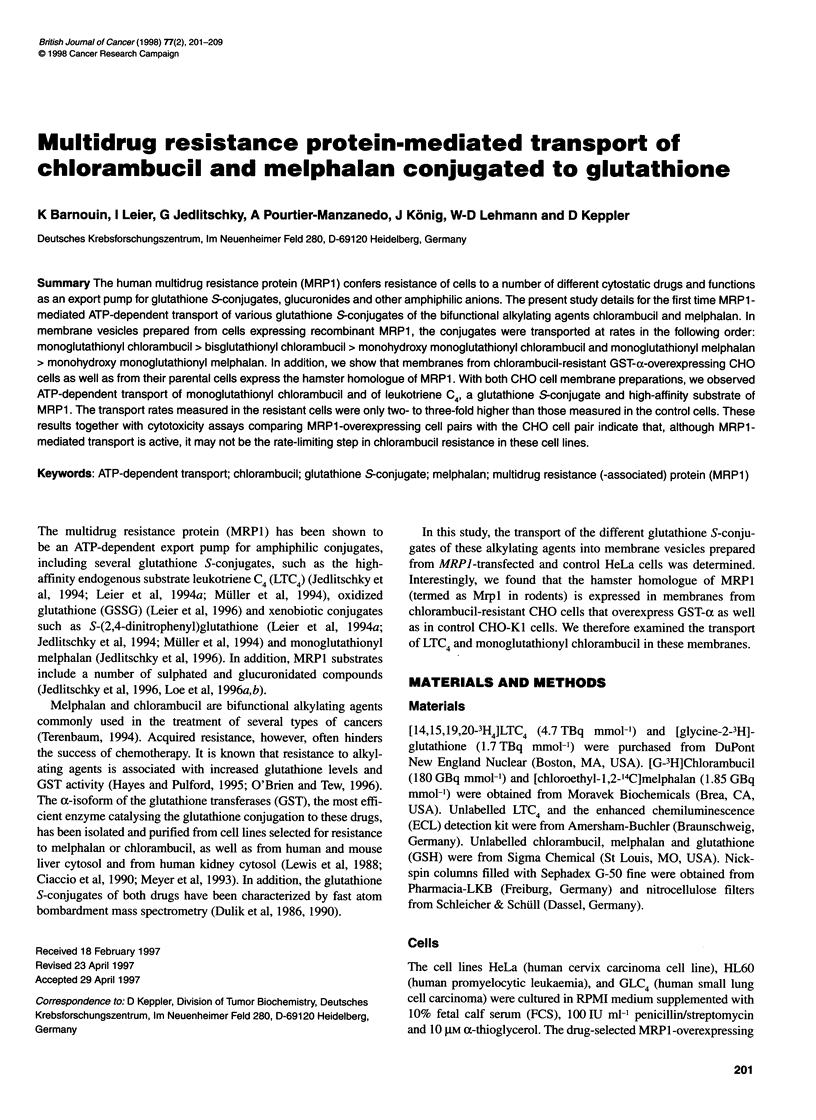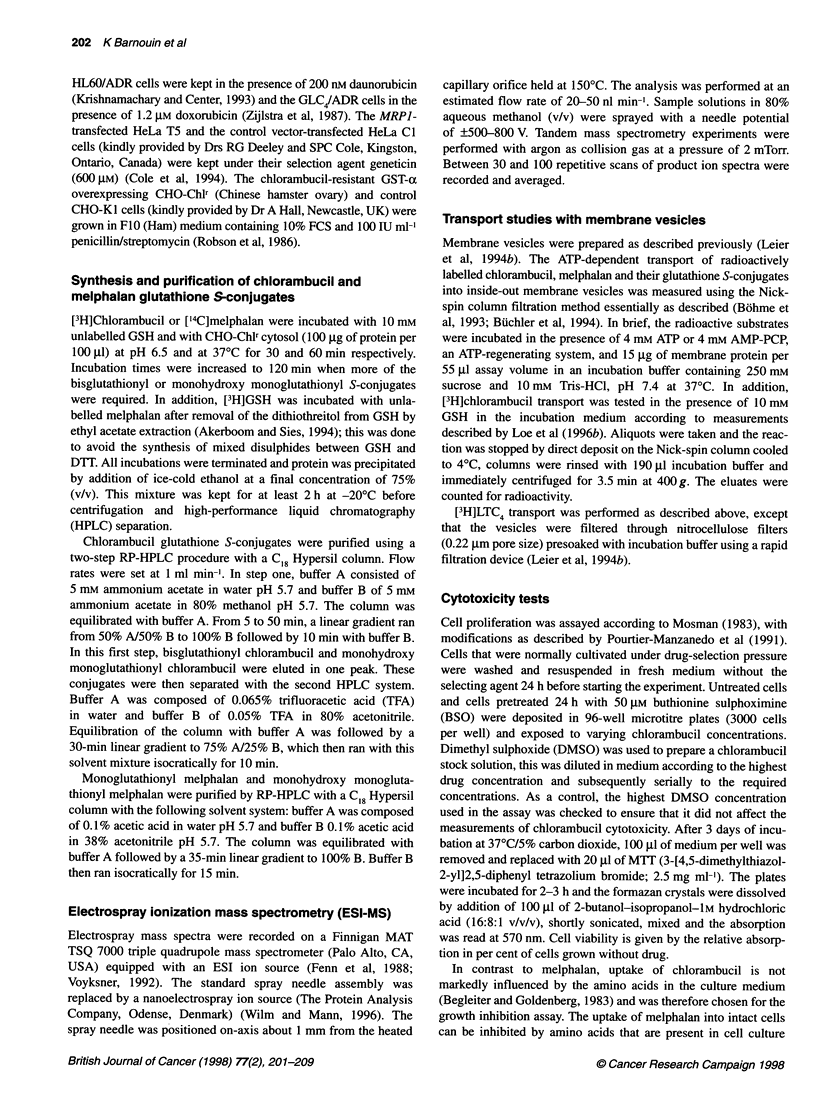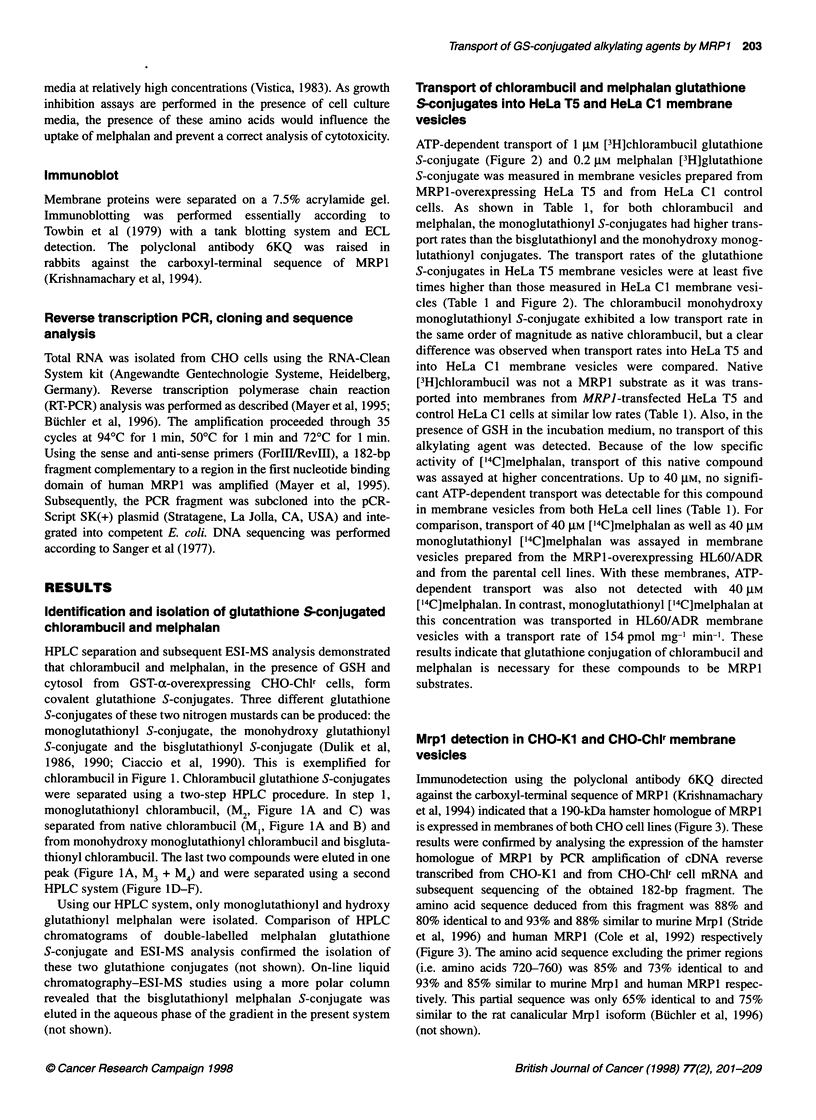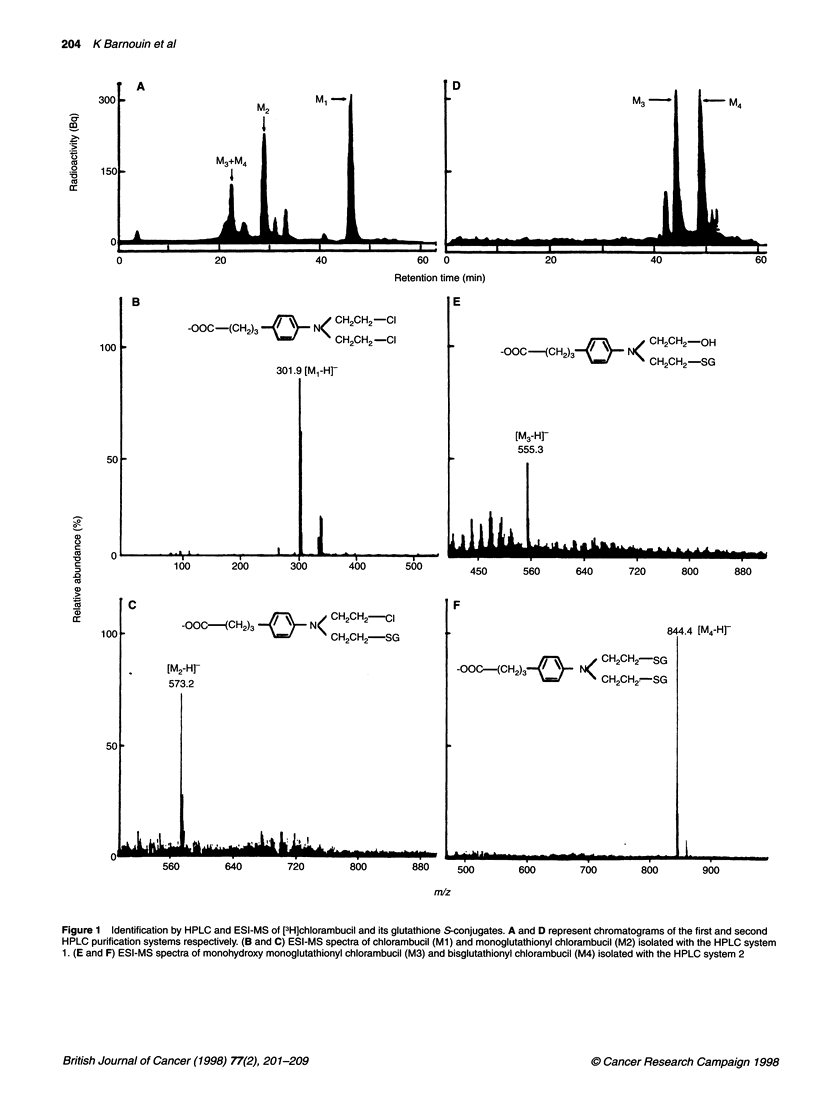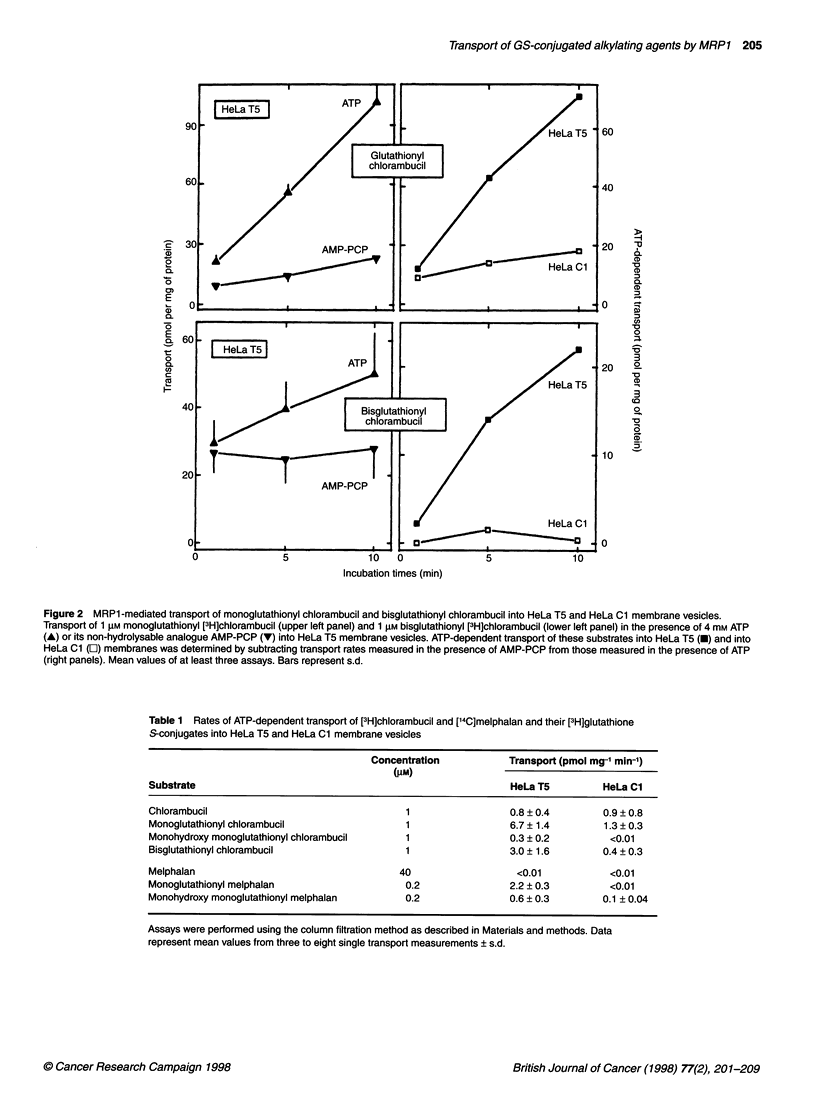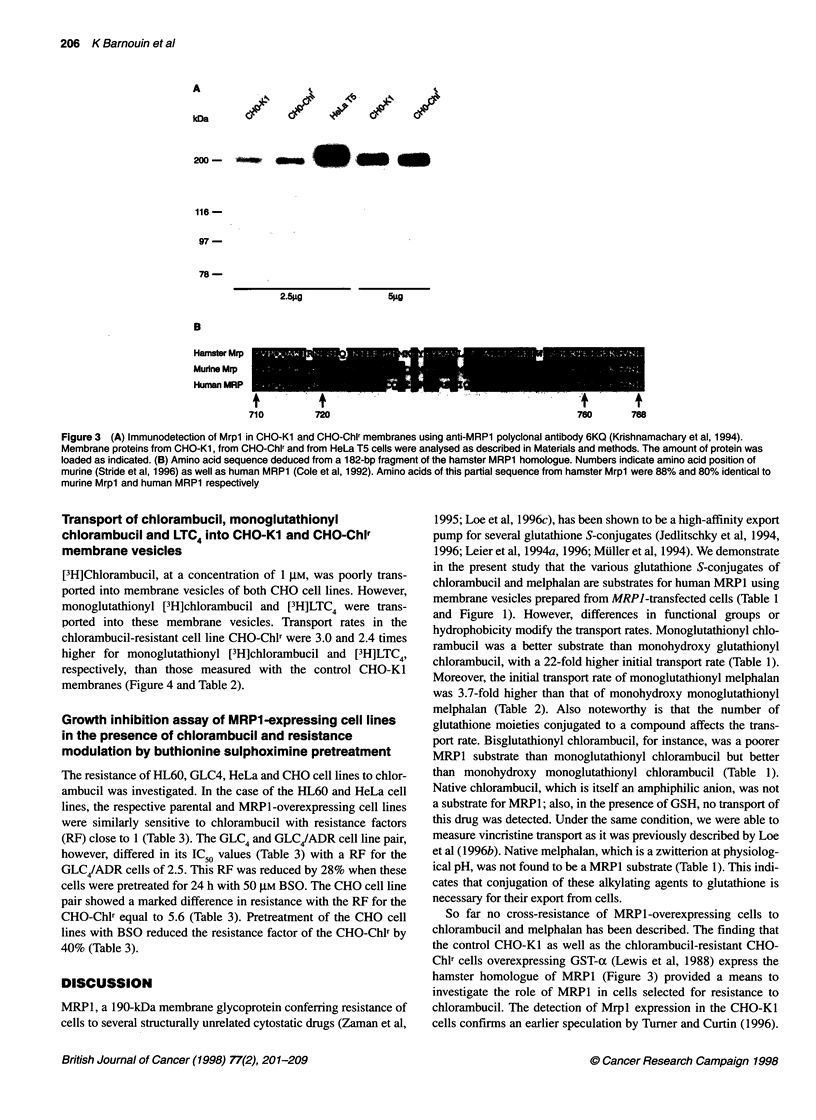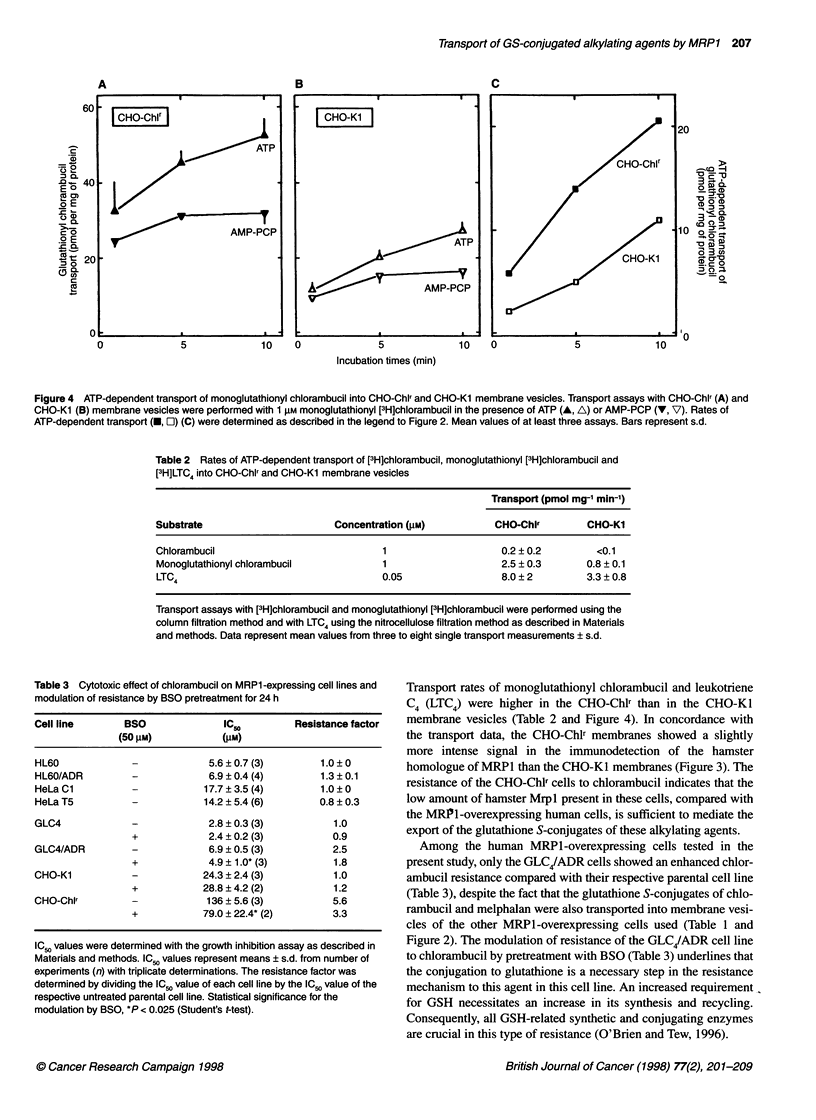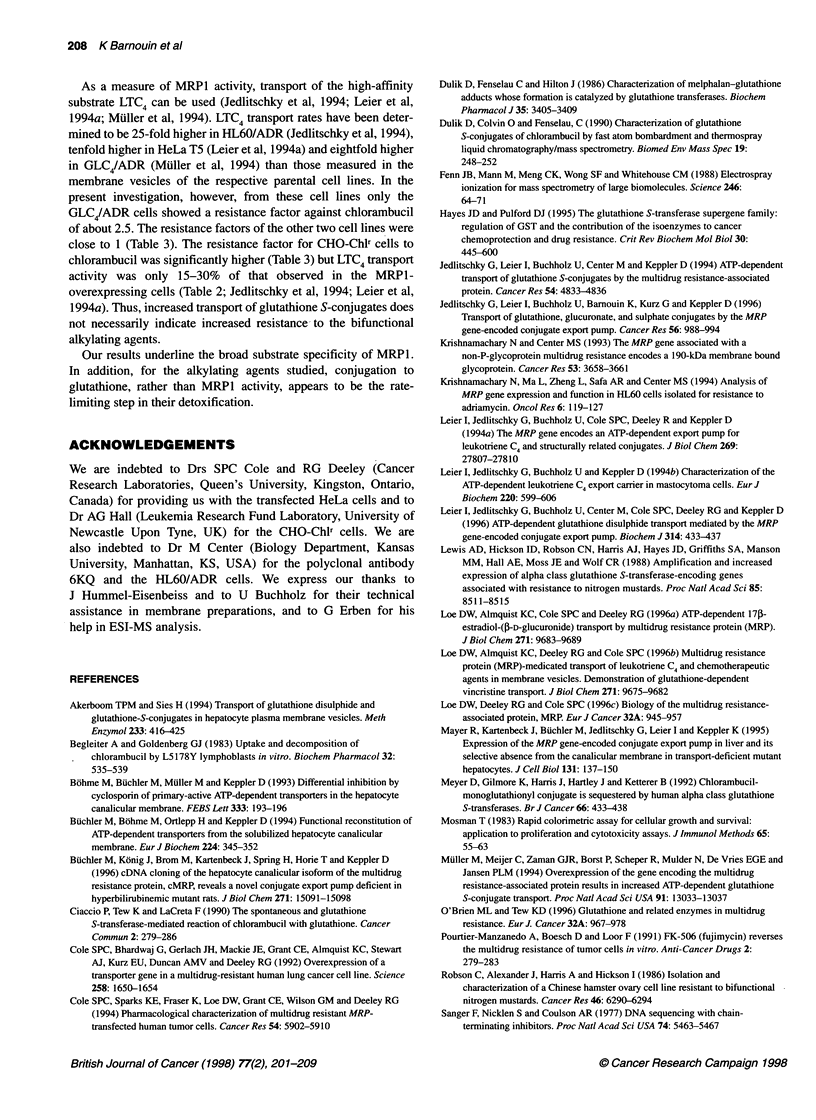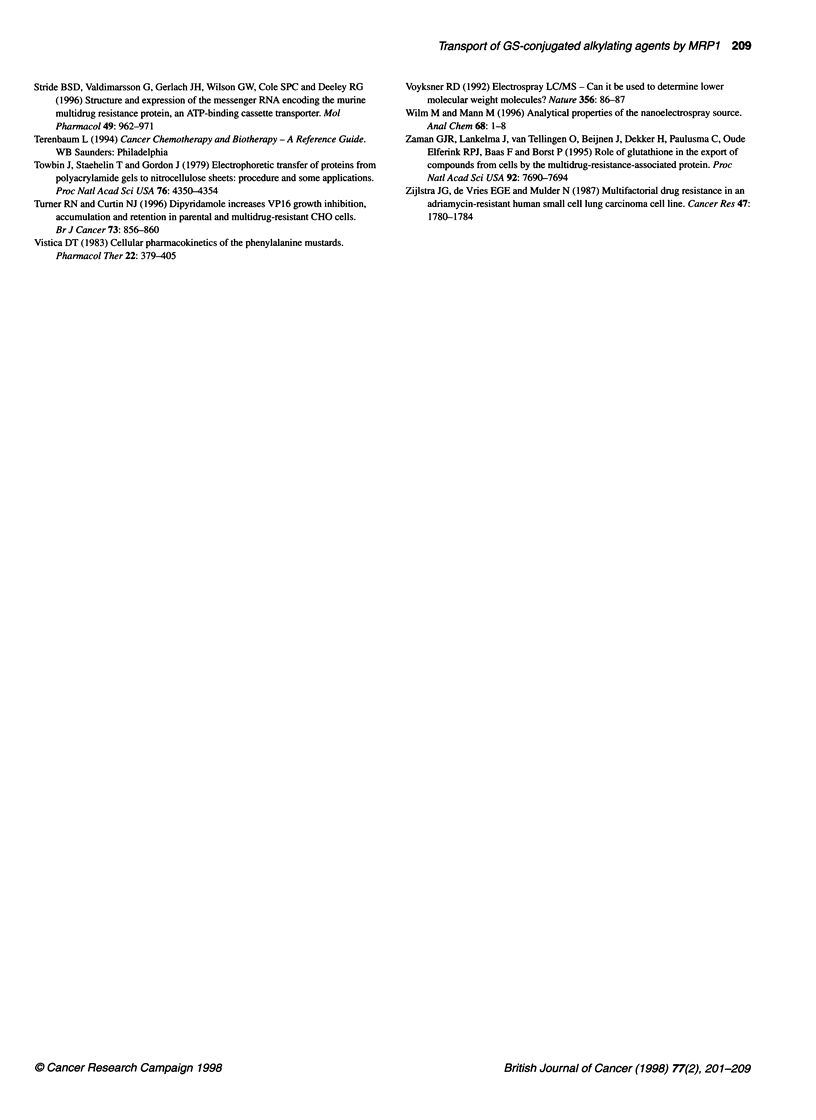# Multidrug resistance protein-mediated transport of chlorambucil and melphalan conjugated to glutathione.

**DOI:** 10.1038/bjc.1998.34

**Published:** 1998

**Authors:** K. Barnouin, I. Leier, G. Jedlitschky, A. Pourtier-Manzanedo, J. KÃ¶nig, W. D. Lehmann, D. Keppler

**Affiliations:** Deutsches Krebsforschungszentrum, Heidelberg, Germany.

## Abstract

**Images:**


British Journal of Cancer (1998) 77(2), 201–209
© 1998 Cancer Research Campaign

### Multidrug resistance protein-mediated transport of chlorambucil and melphalan conjugated to glutathione

**K Barnouin, I Leier, G Jedlitschky, A Pourtier-Manzanedo, J König, W-D Lehmann and D Keppler**

Deutsches Krebsforschungszentrum, Im Neuenheimer Feld 280, D-69120 Heidelberg, Germany

**Summary** The human multidrug resistance protein (MRP1) confers resistance of cells to a number of different cytostatic drugs and functions as an export pump for glutathione *S*-conjugates, glucuronides and other amphiphilic anions. The present study details for the first time MRP1-mediated ATP-dependent transport of various glutathione *S*-conjugates of the bifunctional alkylating agents chlorambucil and melphalan. In membrane vesicles prepared from cells expressing recombinant MRP1, the conjugates were transported at rates in the following order: monoglutathionyl chlorambucil > bisglutathionyl chlorambucil > monohydroxy monoglutathionyl chlorambucil and monoglutathionyl melphalan > monohydroxy monoglutathionyl melphalan. In addition, we show that membranes from chlorambucil-resistant GST-α-overexpressing CHO cells as well as from their parental cells express the hamster homologue of MRP1. With both CHO cell membrane preparations, we observed ATP-dependent transport of monoglutathionyl chlorambucil and of leukotriene $C_4$, a glutathione *S*-conjugate and high-affinity substrate of MRP1. The transport rates measured in the resistant cells were only two- to three-fold higher than those measured in the control cells. These results together with cytotoxicity assays comparing MRP1-overexpressing cell pairs with the CHO cell pair indicate that, although MRP1-mediated transport is active, it may not be the rate-limiting step in chlorambucil resistance in these cell lines.

**Keywords**: ATP-dependent transport; chlorambucil; glutathione *S*-conjugate; melphalan; multidrug resistance (-associated) protein (MRP1)

The multidrug resistance protein (MRP1) has been shown to be an ATP-dependent export pump for amphiphilic conjugates, including several glutathione *S*-conjugates, such as the high-affinity endogenous substrate leukotriene $C_4$ ($LTC_4$) (Jedlitschky et al, 1994; Leier et al, 1994*a*; Müller et al, 1994), oxidized glutathione (GSSG) (Leier et al, 1996) and xenobiotic conjugates such as *S*-(2,4-dinitrophenyl)glutathione (Leier et al, 1994*a*; Jedlitschky et al, 1994; Müller et al, 1994) and monoglutathionyl melphalan (Jedlitschky et al, 1996). In addition, MRP1 substrates include a number of sulphated and glucuronidated compounds (Jedlitschky et al, 1996, Loe et al, 1996*a,b*).

Melphalan and chlorambucil are bifunctional alkylating agents commonly used in the treatment of several types of cancers (Terenbaum, 1994). Acquired resistance, however, often hinders the success of chemotherapy. It is known that resistance to alkylating agents is associated with increased glutathione levels and GST activity (Hayes and Pulford, 1995; O'Brien and Tew, 1996). The α-isoform of the glutathione transferases (GST), the most efficient enzyme catalysing the glutathione conjugation to these drugs, has been isolated and purified from cell lines selected for resistance to melphalan or chlorambucil, as well as from human and mouse liver cytosol and from human kidney cytosol (Lewis et al, 1988; Ciaccio et al, 1990; Meyer et al, 1993). In addition, the glutathione *S*-conjugates of both drugs have been characterized by fast atom bombardment mass spectrometry (Dulik et al, 1986, 1990).

Received 18 February 1997
Revised 23 April 1997
Accepted 29 April 1997

*Correspondence to:* D Keppler, Division of Tumor Biochemistry, Deutsches Krebsforschungszentrum, Im Neuenheimer Feld 280, D-69120 Heidelberg, Germany

In this study, the transport of the different glutathione *S*-conjugates of these alkylating agents into membrane vesicles prepared from *MRP1*-transfected and control HeLa cells was determined. Interestingly, we found that the hamster homologue of MRP1 (termed as Mrp1 in rodents) is expressed in membranes from chlorambucil-resistant CHO cells that overexpress GST-α as well as in control CHO-K1 cells. We therefore examined the transport of $LTC_4$ and monoglutathionyl chlorambucil in these membranes.

### MATERIALS AND METHODS

### Materials

[14,15,19,20-$^3H_4$]$LTC_4$ (4.7 TBq $mmol^{-1}$) and [glycine-2-$^3$H]-glutathione (1.7 TBq $mmol^{-1}$) were purchased from DuPont New England Nuclear (Boston, MA, USA). [G-$^3$H]Chlorambucil (180 GBq $mmol^{-1}$) and [chloroethyl-1,2-$^{14}$C]melphalan (1.85 GBq $mmol^{-1}$) were obtained from Moravek Biochemicals (Brea, CA, USA). Unlabelled $LTC_4$ and the enhanced chemiluminescence (ECL) detection kit were from Amersham-Buchler (Braunschweig, Germany). Unlabelled chlorambucil, melphalan and glutathione (GSH) were from Sigma Chemical (St Louis, MO, USA). Nick-spin columns filled with Sephadex G-50 fine were obtained from Pharmacia-LKB (Freiburg, Germany) and nitrocellulose filters from Schleicher & Schüll (Dassel, Germany).

### Cells

The cell lines HeLa (human cervix carcinoma cell line), HL60 (human promyelocytic leukaemia), and $GLC_4$ (human small lung cell carcinoma) were cultured in RPMI medium supplemented with 10% fetal calf serum (FCS), 100 IU $ml^{-1}$ penicillin/streptomycin and 10 μM α-thioglycerol. The drug-selected MRP1-overexpressing

HL60/ADR cells were kept in the presence of 200 nM daunorubicin (Krishnamachary and Center, 1993) and the $GLC_4$/ADR cells in the presence of 1.2 μM doxorubicin (Zijlstra et al, 1987). The *MRP1*-transfected HeLa T5 and the control vector-transfected HeLa C1 cells (kindly provided by Drs RG Deeley and SPC Cole, Kingston, Ontario, Canada) were kept under their selection agent geneticin (600 μM) (Cole et al, 1994). The chlorambucil-resistant GST-α overexpressing CHO-Chl$^r$ (Chinese hamster ovary) and control CHO-K1 cells (kindly provided by Dr A Hall, Newcastle, UK) were grown in F10 (Ham) medium containing 10% FCS and 100 IU $ml^{-1}$ penicillin/streptomycin (Robson et al, 1986).

### Synthesis and purification of chlorambucil and melphalan glutathione *S*-conjugates

[$^3$H]Chlorambucil or [$^{14}$C]melphalan were incubated with 10 mM unlabelled GSH and with CHO-Chl$^r$ cytosol (100 μg of protein per 100 μl) at pH 6.5 and at 37°C for 30 and 60 min respectively. Incubation times were increased to 120 min when more of the bisglutathionyl or monohydroxy monoglutathionyl *S*-conjugates were required. In addition, [$^3$H]GSH was incubated with unlabelled melphalan after removal of the dithiothreitol from GSH by ethyl acetate extraction (Akerboom and Sies, 1994); this was done to avoid the synthesis of mixed disulphides between GSH and DTT. All incubations were terminated and protein was precipitated by addition of ice-cold ethanol at a final concentration of 75% (v/v). This mixture was kept for at least 2 h at −20°C before centrifugation and high-performance liquid chromatography (HPLC) separation.

Chlorambucil glutathione *S*-conjugates were purified using a two-step RP-HPLC procedure with a $C_{18}$ Hypersil column. Flow rates were set at 1 ml $min^{-1}$. In step one, buffer A consisted of 5 mM ammonium acetate in water pH 5.7 and buffer B of 5 mM ammonium acetate in 80% methanol pH 5.7. The column was equilibrated with buffer A. From 5 to 50 min, a linear gradient ran from 50% A/50% B to 100% B followed by 10 min with buffer B. In this first step, bisglutathionyl chlorambucil and monohydroxy monoglutathionyl chlorambucil were eluted in one peak. These conjugates were then separated with the second HPLC system. Buffer A was composed of 0.065% trifluoracetic acid (TFA) in water and buffer B of 0.05% TFA in 80% acetonitrile. Equilibration of the column with buffer A was followed by a 30-min linear gradient to 75% A/25% B, which then ran with this solvent mixture isocratically for 10 min.

Monoglutathionyl melphalan and monohydroxy monoglutathionyl melphalan were purified by RP-HPLC with a $C_{18}$ Hypersil column with the following solvent system: buffer A was composed of 0.1% acetic acid in water pH 5.7 and buffer B 0.1% acetic acid in 38% acetonitrile pH 5.7. The column was equilibrated with buffer A followed by a 35-min linear gradient to 100% B. Buffer B then ran isocratically for 15 min.

### Electrospray ionization mass spectrometry (ESI-MS)

Electrospray mass spectra were recorded on a Finnigan MAT TSQ 7000 triple quadrupole mass spectrometer (Palo Alto, CA, USA) equipped with an ESI ion source (Fenn et al, 1988; Voyksner, 1992). The standard spray needle assembly was replaced by a nanoelectrospray ion source (The Protein Analysis Company, Odense, Denmark) (Wilm and Mann, 1996). The spray needle was positioned on-axis about 1 mm from the heated capillary orifice held at 150°C. The analysis was performed at an estimated flow rate of 20–50 nl $min^{-1}$. Sample solutions in 80% aqueous methanol (v/v) were sprayed with a needle potential of ±500–800 V. Tandem mass spectrometry experiments were performed with argon as collision gas at a pressure of 2 mTorr. Between 30 and 100 repetitive scans of product ion spectra were recorded and averaged.

### Transport studies with membrane vesicles

Membrane vesicles were prepared as described previously (Leier et al, 1994*b*). The ATP-dependent transport of radioactively labelled chlorambucil, melphalan and their glutathione *S*-conjugates into inside-out membrane vesicles was measured using the Nick-spin column filtration method essentially as described (Böhme et al, 1993; Büchler et al, 1994). In brief, the radioactive substrates were incubated in the presence of 4 mM ATP or 4 mM AMP-PCP, an ATP-regenerating system, and 15 μg of membrane protein per 55 μl assay volume in an incubation buffer containing 250 mM sucrose and 10 mM Tris-HCl, pH 7.4 at 37°C. In addition, [$^3$H]chlorambucil transport was tested in the presence of 10 mM GSH in the incubation medium according to measurements described by Loe et al (1996*b*). Aliquots were taken and the reaction was stopped by direct deposit on the Nick-spin column cooled to 4°C, columns were rinsed with 190 μl incubation buffer and immediately centrifuged for 3.5 min at 400 ***g***. The eluates were counted for radioactivity.

[$^3$H]$LTC_4$ transport was performed as described above, except that the vesicles were filtered through nitrocellulose filters (0.22 μm pore size) presoaked with incubation buffer using a rapid filtration device (Leier et al, 1994*b*).

### Cytotoxicity tests

Cell proliferation was assayed according to Mosman (1983), with modifications as described by Pourtier-Manzanedo et al (1991). Cells that were normally cultivated under drug-selection pressure were washed and resuspended in fresh medium without the selecting agent 24 h before starting the experiment. Untreated cells and cells pretreated 24 h with 50 μM buthionine sulphoximine (BSO) were deposited in 96-well microtitre plates (3000 cells per well) and exposed to varying chlorambucil concentrations. Dimethyl sulphoxide (DMSO) was used to prepare a chlorambucil stock solution, this was diluted in medium according to the highest drug concentration and subsequently serially to the required concentrations. As a control, the highest DMSO concentration used in the assay was checked to ensure that it did not affect the measurements of chlorambucil cytotoxicity. After 3 days of incubation at 37°C/5% carbon dioxide, 100 μl of medium per well was removed and replaced with 20 μl of MTT (3-[4,5-dimethylthiazol-2-yl]2,5-diphenyl tetrazolium bromide; 2.5 mg $ml^{-1}$). The plates were incubated for 2–3 h and the formazan crystals were dissolved by addition of 100 μl of 2-butanol–isopropanol–1M hydrochloric acid (16:8:1 v/v/v), shortly sonicated, mixed and the absorption was read at 570 nm. Cell viability is given by the relative absorption in per cent of cells grown without drug.

In contrast to melphalan, uptake of chlorambucil is not markedly influenced by the amino acids in the culture medium (Begleiter and Goldenberg, 1983) and was therefore chosen for the growth inhibition assay. The uptake of melphalan into intact cells can be inhibited by amino acids that are present in cell culture

© Cancer Research Campaign 1998

media at relatively high concentrations (Vistica, 1983). As growth inhibition assays are performed in the presence of cell culture media, the presence of these amino acids would influence the uptake of melphalan and prevent a correct analysis of cytotoxicity.

### Immunoblot

Membrane proteins were separated on a 7.5% acrylamide gel. Immunoblotting was performed essentially according to Towbin et al (1979) with a tank blotting system and ECL detection. The polyclonal antibody 6KQ was raised in rabbits against the carboxyl-terminal sequence of MRP1 (Krishnamachary et al, 1994).

### Reverse transcription PCR, cloning and sequence analysis

Total RNA was isolated from CHO cells using the RNA-Clean System kit (Angewandte Gentechnologie Systeme, Heidelberg, Germany). Reverse transcription polymerase chain reaction (RT-PCR) analysis was performed as described (Mayer et al, 1995; Büchler et al, 1996). The amplification proceeded through 35 cycles at 94°C for 1 min, 50°C for 1 min and 72°C for 1 min. Using the sense and anti-sense primers (ForIII/RevIII), a 182-bp fragment complementary to a region in the first nucleotide binding domain of human MRP1 was amplified (Mayer et al, 1995). Subsequently, the PCR fragment was subcloned into the pCR-Script SK(+) plasmid (Stratagene, La Jolla, CA, USA) and integrated into competent *E. coli*. DNA sequencing was performed according to Sanger et al (1977).

### RESULTS

### Identification and isolation of glutathione *S*-conjugated chlorambucil and melphalan

HPLC separation and subsequent ESI-MS analysis demonstrated that chlorambucil and melphalan, in the presence of GSH and cytosol from GST-α-overexpressing CHO-Chl$^r$ cells, form covalent glutathione *S*-conjugates. Three different glutathione *S*-conjugates of these two nitrogen mustards can be produced: the monoglutathionyl *S*-conjugate, the monohydroxy glutathionyl *S*-conjugate and the bisglutathionyl *S*-conjugate (Dulik et al, 1986, 1990; Ciaccio et al, 1990). This is exemplified for chlorambucil in Figure 1. Chlorambucil glutathione *S*-conjugates were separated using a two-step HPLC procedure. In step 1, monoglutathionyl chlorambucil, ($M_2$, Figure 1A and C) was separated from native chlorambucil ($M_1$, Figure 1A and B) and from monohydroxy monoglutathionyl chlorambucil and bisglutathionyl chlorambucil. The last two compounds were eluted in one peak (Figure 1A, $M_3$ + $M_4$) and were separated using a second HPLC system (Figure 1D–F).

Using our HPLC system, only monoglutathionyl and hydroxy glutathionyl melphalan were isolated. Comparison of HPLC chromatograms of double-labelled melphalan glutathione *S*-conjugate and ESI-MS analysis confirmed the isolation of these two glutathione conjugates (not shown). On-line liquid chromatography–ESI-MS studies using a more polar column revealed that the bisglutathionyl melphalan *S*-conjugate was eluted in the aqueous phase of the gradient in the present system (not shown).

### Transport of chlorambucil and melphalan glutathione *S*-conjugates into HeLa T5 and HeLa C1 membrane vesicles

ATP-dependent transport of 1 μM [$^3$H]chlorambucil glutathione *S*-conjugate (Figure 2) and 0.2 μM melphalan [$^3$H]glutathione *S*-conjugate was measured in membrane vesicles prepared from MRP1-overexpressing HeLa T5 and from HeLa C1 control cells. As shown in Table 1, for both chlorambucil and melphalan, the monoglutathionyl *S*-conjugates had higher transport rates than the bisglutathionyl and the monohydroxy monoglutathionyl conjugates. The transport rates of the glutathione *S*-conjugates in HeLa T5 membrane vesicles were at least five times higher than those measured in HeLa C1 membrane vesicles (Table 1 and Figure 2). The chlorambucil monohydroxy monoglutathionyl *S*-conjugate exhibited a low transport rate in the same order of magnitude as native chlorambucil, but a clear difference was observed when transport rates into HeLa T5 and into HeLa C1 membrane vesicles were compared. Native [$^3$H]chlorambucil was not a MRP1 substrate as it was transported into membranes from *MRP1*-transfected HeLa T5 and control HeLa C1 cells at similar low rates (Table 1). Also, in the presence of GSH in the incubation medium, no transport of this alkylating agent was detected. Because of the low specific activity of [$^{14}$C]melphalan, transport of this native compound was assayed at higher concentrations. Up to 40 μM, no significant ATP-dependent transport was detectable for this compound in membrane vesicles from both HeLa cell lines (Table 1). For comparison, transport of 40 μM [$^{14}$C]melphalan as well as 40 μM monoglutathionyl [$^{14}$C]melphalan was assayed in membrane vesicles prepared from the MRP1-overexpressing HL60/ADR and from the parental cell lines. With these membranes, ATP-dependent transport was also not detected with 40 μM [$^{14}$C]melphalan. In contrast, monoglutathionyl [$^{14}$C]melphalan at this concentration was transported in HL60/ADR membrane vesicles with a transport rate of 154 pmol mg$^{-1}$ min$^{-1}$. These results indicate that glutathione conjugation of chlorambucil and melphalan is necessary for these compounds to be MRP1 substrates.

### Mrp1 detection in CHO-K1 and CHO-Chl$^r$ membrane vesicles

Immunodetection using the polyclonal antibody 6KQ directed against the carboxyl-terminal sequence of MRP1 (Krishnamachary et al, 1994) indicated that a 190-kDa hamster homologue of MRP1 is expressed in membranes of both CHO cell lines (Figure 3). These results were confirmed by analysing the expression of the hamster homologue of MRP1 by PCR amplification of cDNA reverse transcribed from CHO-K1 and from CHO-Chl$^r$ cell mRNA and subsequent sequencing of the obtained 182-bp fragment. The amino acid sequence deduced from this fragment was 88% and 80% identical to and 93% and 88% similar to murine Mrp1 (Stride et al, 1996) and human MRP1 (Cole et al, 1992) respectively (Figure 3). The amino acid sequence excluding the primer regions (i.e. amino acids 720–760) was 85% and 73% identical to and 93% and 85% similar to murine Mrp1 and human MRP1 respectively. This partial sequence was only 65% identical to and 75% similar to the rat canalicular Mrp1 isoform (Büchler et al, 1996) (not shown).

© Cancer Research Campaign 1998

**Figure 1** Identification by HPLC and ESI-MS of [$^3$H]chlorambucil and its glutathione *S*-conjugates. **A** and **D** represent chromatograms of the first and second HPLC purification systems respectively. (**B** and **C**) ESI-MS spectra of chlorambucil (M1) and monoglutathionyl chlorambucil (M2) isolated with the HPLC system 1. (**E** and **F**) ESI-MS spectra of monohydroxy monoglutathionyl chlorambucil (M3) and bisglutathionyl chlorambucil (M4) isolated with the HPLC system 2

© Cancer Research Campaign 1998

**Figure 2** MRP1-mediated transport of monoglutathionyl chlorambucil and bisglutathionyl chlorambucil into HeLa T5 and HeLa C1 membrane vesicles. Transport of 1 μM monoglutathionyl [$^3$H]chlorambucil (upper left panel) and 1 μM bisglutathionyl [$^3$H]chlorambucil (lower left panel) in the presence of 4 mM ATP (▲) or its non-hydrolysable analogue AMP-PCP (▼) into HeLa T5 membrane vesicles. ATP-dependent transport of these substrates into HeLa T5 (■) and into HeLa C1 (□) membranes was determined by subtracting transport rates measured in the presence of AMP-PCP from those measured in the presence of ATP (right panels). Mean values of at least three assays. Bars represent s.d.

**Table 1** Rates of ATP-dependent transport of [$^3$H]chlorambucil and [$^{14}$C]melphalan and their [$^3$H]glutathione *S*-conjugates into HeLa T5 and HeLa C1 membrane vesicles

| Substrate | Concentration (μM) | Transport (pmol mg$^{-1}$ min$^{-1}$) HeLa T5 | HeLa C1 |
|---|---|---|---|
| Chlorambucil | 1 | 0.8 ± 0.4 | 0.9 ± 0.8 |
| Monoglutathionyl chlorambucil | 1 | 6.7 ± 1.4 | 1.3 ± 0.3 |
| Monohydroxy monoglutathionyl chlorambucil | 1 | 0.3 ± 0.2 | <0.01 |
| Bisglutathionyl chlorambucil | 1 | 3.0 ± 1.6 | 0.4 ± 0.3 |
| Melphalan | 40 | <0.01 | <0.01 |
| Monoglutathionyl melphalan | 0.2 | 2.2 ± 0.3 | <0.01 |
| Monohydroxy monoglutathionyl melphalan | 0.2 | 0.6 ± 0.3 | 0.1 ± 0.04 |

Assays were performed using the column filtration method as described in Materials and methods. Data represent mean values from three to eight single transport measurements ± s.d.

© Cancer Research Campaign 1998

**Figure 3** (**A**) Immunodetection of Mrp1 in CHO-K1 and CHO-Chl$^r$ membranes using anti-MRP1 polyclonal antibody 6KQ (Krishnamachary et al, 1994). Membrane proteins from CHO-K1, from CHO-Chl$^r$ and from HeLa T5 cells were analysed as described in Materials and methods. The amount of protein was loaded as indicated. (**B**) Amino acid sequence deduced from a 182-bp fragment of the hamster MRP1 homologue. Numbers indicate amino acid position of murine (Stride et al, 1996) as well as human MRP1 (Cole et al, 1992). Amino acids of this partial sequence from hamster Mrp1 were 88% and 80% identical to murine Mrp1 and human MRP1 respectively

### Transport of chlorambucil, monoglutathionyl chlorambucil and $LTC_4$ into CHO-K1 and CHO-Chl$^r$ membrane vesicles

[$^3$H]Chlorambucil, at a concentration of 1 μM, was poorly transported into membrane vesicles of both CHO cell lines. However, monoglutathionyl [$^3$H]chlorambucil and [$^3$H]$LTC_4$ were transported into these membrane vesicles. Transport rates in the chlorambucil-resistant cell line CHO-Chl$^r$ were 3.0 and 2.4 times higher for monoglutathionyl [$^3$H]chlorambucil and [$^3$H]$LTC_4$, respectively, than those measured with the control CHO-K1 membranes (Figure 4 and Table 2).

### Growth inhibition assay of MRP1-expressing cell lines in the presence of chlorambucil and resistance modulation by buthionine sulphoximine pretreatment

The resistance of HL60, GLC4, HeLa and CHO cell lines to chlorambucil was investigated. In the case of the HL60 and HeLa cell lines, the respective parental and MRP1-overexpressing cell lines were similarly sensitive to chlorambucil with resistance factors (RF) close to 1 (Table 3). The $GLC_4$ and $GLC_4$/ADR cell line pair, however, differed in its $IC_{50}$ values (Table 3) with a RF for the $GLC_4$/ADR cells of 2.5. This RF was reduced by 28% when these cells were pretreated for 24 h with 50 μM BSO. The CHO cell line pair showed a marked difference in resistance with the RF for the CHO-Chl$^r$ equal to 5.6 (Table 3). Pretreatment of the CHO cell lines with BSO reduced the resistance factor of the CHO-Chl$^r$ by 40% (Table 3).

### DISCUSSION

MRP1, a 190-kDa membrane glycoprotein conferring resistance of cells to several structurally unrelated cytostatic drugs (Zaman et al, 1995; Loe et al, 1996*c*), has been shown to be a high-affinity export pump for several glutathione *S*-conjugates (Jedlitschky et al, 1994, 1996; Leier et al, 1994*a*, 1996; Müller et al, 1994). We demonstrate in the present study that the various glutathione *S*-conjugates of chlorambucil and melphalan are substrates for human MRP1 using membrane vesicles prepared from *MRP1*-transfected cells (Table 1 and Figure 1). However, differences in functional groups or hydrophobicity modify the transport rates. Monoglutathionyl chlorambucil was a better substrate than monohydroxy glutathionyl chlorambucil, with a 22-fold higher initial transport rate (Table 1). Moreover, the initial transport rate of monoglutathionyl melphalan was 3.7-fold higher than that of monohydroxy monoglutathionyl melphalan (Table 2). Also noteworthy is that the number of glutathione moieties conjugated to a compound affects the transport rate. Bisglutathionyl chlorambucil, for instance, was a poorer MRP1 substrate than monoglutathionyl chlorambucil but better than monohydroxy monoglutathionyl chlorambucil (Table 1). Native chlorambucil, which is itself an amphiphilic anion, was not a substrate for MRP1; also, in the presence of GSH, no transport of this drug was detected. Under the same condition, we were able to measure vincristine transport as it was previously described by Loe et al (1996*b*). Native melphalan, which is a zwitterion at physiological pH, was not found to be a MRP1 substrate (Table 1). This indicates that conjugation of these alkylating agents to glutathione is necessary for their export from cells.

So far no cross-resistance of MRP1-overexpressing cells to chlorambucil and melphalan has been described. The finding that the control CHO-K1 as well as the chlorambucil-resistant CHO-Chl$^r$ cells overexpressing GST-α (Lewis et al, 1988) express the hamster homologue of MRP1 (Figure 3) provided a means to investigate the role of MRP1 in cells selected for resistance to chlorambucil. The detection of Mrp1 expression in the CHO-K1 cells confirms an earlier speculation by Turner and Curtin (1996).

© Cancer Research Campaign 1998

**Figure 4** ATP-dependent transport of monoglutathionyl chlorambucil into CHO-Chl$^r$ and CHO-K1 membrane vesicles. Transport assays with CHO-Chl$^r$ (**A**) and CHO-K1 (**B**) membrane vesicles were performed with 1 μM monoglutathionyl [$^3$H]chlorambucil in the presence of ATP (▲, △) or AMP-PCP (▼, ▽). Rates of ATP-dependent transport (■, □) (**C**) were determined as described in the legend to Figure 2. Mean values of at least three assays. Bars represent s.d.

**Table 2** Rates of ATP-dependent transport of [$^3$H]chlorambucil, monoglutathionyl [$^3$H]chlorambucil and [$^3$H]LTC$_4$ into CHO-Chl$^r$ and CHO-K1 membrane vesicles

| | | Transport (pmol mg$^{-1}$ min$^{-1}$) | |
|---|---|---|---|
| **Substrate** | **Concentration (μM)** | **CHO-Chl$^r$** | **CHO-K1** |
| Chlorambucil | 1 | 0.2 ± 0.2 | <0.1 |
| Monoglutathionyl chlorambucil | 1 | 2.5 ± 0.3 | 0.8 ± 0.1 |
| LTC$_4$ | 0.05 | 8.0 ± 2 | 3.3 ± 0.8 |

Transport assays with [$^3$H]chlorambucil and monoglutathionyl [$^3$H]chlorambucil were performed using the column filtration method and with LTC$_4$ using the nitrocellulose filtration method as described in Materials and methods. Data represent mean values from three to eight single transport measurements ± s.d.

**Table 3** Cytotoxic effect of chlorambucil on MRP1-expressing cell lines and modulation of resistance by BSO pretreatment for 24 h

| Cell line | BSO (50 μM) | IC$_{50}$ (μM) | Resistance factor |
|---|---|---|---|
| HL60 | – | 5.6 ± 0.7 (3) | 1.0 ± 0 |
| HL60/ADR | – | 6.9 ± 0.4 (4) | 1.3 ± 0.1 |
| HeLa C1 | – | 17.7 ± 3.5 (4) | 1.0 ± 0 |
| HeLa T5 | – | 14.2 ± 5.4 (6) | 0.8 ± 0.3 |
| GLC4 | – | 2.8 ± 0.3 (3) | 1.0 |
| | + | 2.4 ± 0.2 (3) | 0.9 |
| GLC4/ADR | – | 6.9 ± 0.5 (3) | 2.5 |
| | + | 4.9 ± 1.0* (3) | 1.8 |
| CHO-K1 | – | 24.3 ± 2.4 (3) | 1.0 |
| | + | 28.8 ± 4.2 (2) | 1.2 |
| CHO-Chl$^r$ | – | 136 ± 5.6 (3) | 5.6 |
| | + | 79.0 ± 22.4* (2) | 3.3 |

IC$_{50}$ values were determined with the growth inhibition assay as described in Materials and methods. IC$_{50}$ values represent means ± s.d. from number of experiments (*n*) with triplicate determinations. The resistance factor was determined by dividing the IC$_{50}$ value of each cell line by the IC$_{50}$ value of the respective untreated parental cell line. Statistical significance for the modulation by BSO, *$P < 0.025$ (Student's *t*-test).

Transport rates of monoglutathionyl chlorambucil and leukotriene C$_4$ (LTC$_4$) were higher in the CHO-Chl$^r$ than in the CHO-K1 membrane vesicles (Table 2 and Figure 4). In concordance with the transport data, the CHO-Chl$^r$ membranes showed a slightly more intense signal in the immunodetection of the hamster homologue of MRP1 than the CHO-K1 membranes (Figure 3). The resistance of the CHO-Chl$^r$ cells to chlorambucil indicates that the low amount of hamster Mrp1 present in these cells, compared with the MRP1-overexpressing human cells, is sufficient to mediate the export of the glutathione *S*-conjugates of these alkylating agents.

Among the human MRP1-overexpressing cells tested in the present study, only the GLC$_4$/ADR cells showed an enhanced chlorambucil resistance compared with their respective parental cell line (Table 3), despite the fact that the glutathione *S*-conjugates of chlorambucil and melphalan were also transported into membrane vesicles of the other MRP1-overexpressing cells used (Table 1 and Figure 2). The modulation of resistance of the GLC$_4$/ADR cell line to chlorambucil by pretreatment with BSO (Table 3) underlines that the conjugation to glutathione is a necessary step in the resistance mechanism to this agent in this cell line. An increased requirement for GSH necessitates an increase in its synthesis and recycling. Consequently, all GSH-related synthetic and conjugating enzymes are crucial in this type of resistance (O'Brien and Tew, 1996).

As a measure of MRP1 activity, transport of the high-affinity substrate $LTC_4$ can be used (Jedlitschky et al, 1994; Leier et al, 1994*a*; Müller et al, 1994). $LTC_4$ transport rates have been determined to be 25-fold higher in HL60/ADR (Jedlitschky et al, 1994), tenfold higher in HeLa T5 (Leier et al, 1994a) and eightfold higher in $GLC_4$/ADR (Müller et al, 1994) than those measured in the membrane vesicles of the respective parental cell lines. In the present investigation, however, from these cell lines only the $GLC_4$/ADR cells showed a resistance factor against chlorambucil of about 2.5. The resistance factors of the other two cell lines were close to 1 (Table 3). The resistance factor for CHO-Chl$^r$ cells to chlorambucil was significantly higher (Table 3) but $LTC_4$ transport activity was only 15–30% of that observed in the MRP1-overexpressing cells (Table 2; Jedlitschky et al, 1994; Leier et al, 1994*a*). Thus, increased transport of glutathione *S*-conjugates does not necessarily indicate increased resistance to the bifunctional alkylating agents.

Our results underline the broad substrate specificity of MRP1. In addition, for the alkylating agents studied, conjugation to glutathione, rather than MRP1 activity, appears to be the rate-limiting step in their detoxification.

### ACKNOWLEDGEMENTS

We are indebted to Drs SPC Cole and RG Deeley (Cancer Research Laboratories, Queen's University, Kingston, Ontario, Canada) for providing us with the transfected HeLa cells and to Dr AG Hall (Leukemia Research Fund Laboratory, University of Newcastle Upon Tyne, UK) for the CHO-Chl$^r$ cells. We are also indebted to Dr M Center (Biology Department, Kansas University, Manhattan, KS, USA) for the polyclonal antibody 6KQ and the HL60/ADR cells. We express our thanks to J Hummel-Eisenbeiss and to U Buchholz for their technical assistance in membrane preparations, and to G Erben for his help in ESI-MS analysis.

### REFERENCES

Akerboom TPM and Sies H (1994) Transport of glutathione disulphide and glutathione-*S*-conjugates in hepatocyte plasma membrane vesicles. *Meth Enzymol* **233**: 416–425

Begleiter A and Goldenberg GJ (1983) Uptake and decomposition of chlorambucil by L5178Y lymphoblasts *in vitro*. *Biochem Pharmacol* **32**: 535–539

Böhme M, Büchler M, Müller M and Keppler D (1993) Differential inhibition by cyclosporin of primary-active ATP-dependent transporters in the hepatocyte canalicular membrane. *FEBS Lett* **333**: 193–196

Büchler M, Böhme M, Ortlepp H and Keppler D (1994) Functional reconstitution of ATP-dependent transporters from the solubilized hepatocyte canalicular membrane. *Eur J Biochem* **224**: 345–352

Büchler M, König J, Brom M, Kartenbeck J, Spring H, Horie T and Keppler D (1996) cDNA cloning of the hepatocyte canalicular isoform of the multidrug resistance protein, cMRP, reveals a novel conjugate export pump deficient in hyperbilirubinemic mutant rats. *J Biol Chem* **271**: 15091–15098

Ciaccio P, Tew K and LaCreta F (1990) The spontaneous and glutathione *S*-transferase-mediated reaction of chlorambucil with glutathione. *Cancer Commun* **2**: 279–286

Cole SPC, Bhardwaj G, Gerlach JH, Mackie JE, Grant CE, Almquist KC, Stewart AJ, Kurz EU, Duncan AMV and Deeley RG (1992) Overexpression of a transporter gene in a multidrug-resistant human lung cancer cell line. *Science* **258**: 1650–1654

Cole SPC, Sparks KE, Fraser K, Loe DW, Grant CE, Wilson GM and Deeley RG (1994) Pharmacological characterization of multidrug resistant *MRP*-transfected human tumor cells. *Cancer Res* **54**: 5902–5910

Dulik D, Fenselau C and Hilton J (1986) Characterization of melphalan–glutathione adducts whose formation is catalyzed by glutathione transferases. *Biochem Pharmacol J* **35**: 3405–3409

Dulik D, Colvin O and Fenselau, C (1990) Characterization of glutathione *S*-conjugates of chlorambucil by fast atom bombardment and thermospray liquid chromatography/mass spectrometry. *Biomed Env Mass Spec* **19**: 248–252

Fenn JB, Mann M, Meng CK, Wong SF and Whitehouse CM (1988) Electrospray ionization for mass spectrometry of large biomolecules. *Science* **246**: 64–71

Hayes JD and Pulford DJ (1995) The glutathione *S*-transferase supergene family: regulation of GST and the contribution of the isoenzymes to cancer chemoprotection and drug resistance. *Crit Rev Biochem Mol Biol* **30**: 445–600

Jedlitschky G, Leier I, Buchholz U, Center M and Keppler D (1994) ATP-dependent transport of glutathione *S*-conjugates by the multidrug resistance-associated protein. *Cancer Res* **54**: 4833–4836

Jedlitschky G, Leier I, Buchholz U, Barnouin K, Kurz G and Keppler D (1996) Transport of glutathione, glucuronate, and sulphate conjugates by the *MRP* gene-encoded conjugate export pump. *Cancer Res* **56**: 988–994

Krishnamachary N and Center MS (1993) The *MRP* gene associated with a non-P-glycoprotein multidrug resistance encodes a 190-kDa membrane bound glycoprotein. *Cancer Res* **53**: 3658–3661

Krishnamachary N, Ma L, Zheng L, Safa AR and Center MS (1994) Analysis of *MRP* gene expression and function in HL60 cells isolated for resistance to adriamycin. *Oncol Res* **6**: 119–127

Leier I, Jedlitschky G, Buchholz U, Cole SPC, Deeley R and Keppler D (1994*a*) The *MRP* gene encodes an ATP-dependent export pump for leukotriene $C_4$ and structurally related conjugates. *J Biol Chem* **269**: 27807–27810

Leier I, Jedlitschky G, Buchholz U and Keppler D (1994*b*) Characterization of the ATP-dependent leukotriene $C_4$ export carrier in mastocytoma cells. *Eur J Biochem* **220**: 599–606

Leier I, Jedlitschky G, Buchholz U, Center M, Cole SPC, Deeley RG and Keppler D (1996) ATP-dependent glutathione disulphide transport mediated by the *MRP* gene-encoded conjugate export pump. *Biochem J* **314**: 433–437

Lewis AD, Hickson ID, Robson CN, Harris AJ, Hayes JD, Griffiths SA, Manson MM, Hall AE, Moss JE and Wolf CR (1988) Amplification and increased expression of alpha class glutathione *S*-transferase-encoding genes associated with resistance to nitrogen mustards. *Proc Natl Acad Sci* **85**: 8511–8515

Loe DW, Almquist KC, Cole SPC and Deeley RG (1996*a*) ATP-dependent 17β-estradiol-(β-D-glucuronide) transport by multidrug resistance protein (MRP). *J Biol Chem* **271**: 9683–9689

Loe DW, Almquist KC, Deeley RG and Cole SPC (1996*b*) Multidrug resistance protein (MRP)-medicated transport of leukotriene $C_4$ and chemotherapeutic agents in membrane vesicles. Demonstration of glutathione-dependent vincristine transport. *J Biol Chem* **271**: 9675–9682

Loe DW, Deeley RG and Cole SPC (1996*c*) Biology of the multidrug resistance-associated protein, MRP. *Eur J Cancer* **32A**: 945–957

Mayer R, Kartenbeck J, Büchler M, Jedlitschky G, Leier I and Keppler K (1995) Expression of the *MRP* gene-encoded conjugate export pump in liver and its selective absence from the canalicular membrane in transport-deficient mutant hepatocytes. *J Cell Biol* **131**: 137–150

Meyer D, Gilmore K, Harris J, Hartley J and Ketterer B (1992) Chlorambucil-monoglutathionyl conjugate is sequestered by human alpha class glutathione *S*-transferases. *Br J Cancer* **66**: 433–438

Mosman T (1983) Rapid colorimetric assay for cellular growth and survival: application to proliferation and cytotoxicity assays. *J Immunol Methods* **65**: 55–63

Müller M, Meijer C, Zaman GJR, Borst P, Scheper R, Mulder N, De Vries EGE and Jansen PLM (1994) Overexpression of the gene encoding the multidrug resistance-associated protein results in increased ATP-dependent glutathione *S*-conjugate transport. *Proc Natl Acad Sci USA* **91**: 13033–13037

O'Brien ML and Tew KD (1996) Glutathione and related enzymes in multidrug resistance. *Eur J. Cancer* **32A**: 967–978

Pourtier-Manzanedo A, Boesch D and Loor F (1991) FK-506 (fujimycin) reverses the multidrug resistance of tumor cells *in vitro*. *Anti-Cancer Drugs* **2**: 279–283

Robson C, Alexander J, Harris A and Hickson I (1986) Isolation and characterization of a Chinese hamster ovary cell line resistant to bifunctional nitrogen mustards. *Cancer Res* **46**: 6290–6294

Sanger F, Nicklen S and Coulson AR (1977) DNA sequencing with chain-terminating inhibitors. *Proc Natl Acad Sci USA* **74**: 5463–5467

© Cancer Research Campaign 1998

Stride BSD, Valdimarsson G, Gerlach JH, Wilson GW, Cole SPC and Deeley RG (1996) Structure and expression of the messenger RNA encoding the murine multidrug resistance protein, an ATP-binding cassette transporter. *Mol Pharmacol* **49**: 962–971

Terenbaum L (1994) *Cancer Chemotherapy and Biotherapy – A Reference Guide*. WB Saunders: Philadelphia

Towbin J, Staehelin T and Gordon J (1979) Electrophoretic transfer of proteins from polyacrylamide gels to nitrocellulose sheets: procedure and some applications. *Proc Natl Acad Sci USA* **76**: 4350–4354

Turner RN and Curtin NJ (1996) Dipyridamole increases VP16 growth inhibition, accumulation and retention in parental and multidrug-resistant CHO cells. *Br J Cancer* **73**: 856–860

Vistica DT (1983) Cellular pharmacokinetics of the phenylalanine mustards. *Pharmacol Ther* **22**: 379–405

Voyksner RD (1992) Electrospray LC/MS – Can it be used to determine lower molecular weight molecules? *Nature* **356**: 86–87

Wilm M and Mann M (1996) Analytical properties of the nanoelectrospray source. *Anal Chem* **68**: 1–8

Zaman GJR, Lankelma J, van Tellingen O, Beijnen J, Dekker H, Paulusma C, Oude Elferink RPJ, Baas F and Borst P (1995) Role of glutathione in the export of compounds from cells by the multidrug-resistance-associated protein. *Proc Natl Acad Sci USA* **92**: 7690–7694

Zijlstra JG, de Vries EGE and Mulder N (1987) Multifactorial drug resistance in an adriamycin-resistant human small cell lung carcinoma cell line. *Cancer Res* **47**: 1780–1784

© Cancer Research Campaign 1998